# Diagnosing Viral Infections Through T-Cell Receptor Sequencing of Activated CD8^+^ T Cells

**DOI:** 10.1093/infdis/jiad430

**Published:** 2023-10-03

**Authors:** Alexandra Vujkovic, My Ha, Tessa de Block, Lida van Petersen, Isabel Brosius, Caroline Theunissen, Sabrina H van Ierssel, Esther Bartholomeus, Wim Adriaensen, Guido Vanham, George Elias, Pierre Van Damme, Viggo Van Tendeloo, Philippe Beutels, Maartje van Frankenhuijsen, Erika Vlieghe, Benson Ogunjimi, Kris Laukens, Pieter Meysman, Koen Vercauteren

**Affiliations:** Clinical Virology Unit, Department of Clinical Sciences, Institute of Tropical Medicine, Antwerp, Belgium; Antwerp Unit for Data Analysis and Computation in Immunology and Sequencing (AUDACIS), University of Antwerp, Antwerp, Belgium; Adrem Data Lab, Department of Computer Science, University of Antwerp, Antwerp, Belgium; Antwerp Unit for Data Analysis and Computation in Immunology and Sequencing (AUDACIS), University of Antwerp, Antwerp, Belgium; Antwerp Center for Translational Immunology and Virology (ACTIV), Antwerp, Belgium; Centre for Health Economics Research and Modeling Infectious Diseases (CHERMID), University of Antwerp, Belgium; Vaccine and Infectious Disease Institute, University of Antwerp, Belgium; Department of Clinical Sciences, Institute of Tropical Medicine, Antwerp, Belgium; Department of Clinical Sciences, Institute of Tropical Medicine, Antwerp, Belgium; Department of Clinical Sciences, Institute of Tropical Medicine, Antwerp, Belgium; Department of Clinical Sciences, Institute of Tropical Medicine, Antwerp, Belgium; Department of General Internal Medicine, Infectious Diseases and Tropical Medicine, University Hospital Antwerp, Belgium; Antwerp Unit for Data Analysis and Computation in Immunology and Sequencing (AUDACIS), University of Antwerp, Antwerp, Belgium; Antwerp Center for Translational Immunology and Virology (ACTIV), Antwerp, Belgium; Clinical Immunology Unit, Department of Clinical Sciences, Institute of Tropical Medicine, Antwerp, Belgium; Biomedical Department, Institute of Tropical Medicine, Antwerp, Belgium; Laboratory of Experimental Hematology, Faculty of Medicine and Health Sciences, University of Antwerp, Belgium; Vaccine and Infectious Disease Institute, University of Antwerp, Belgium; Laboratory of Experimental Hematology, Faculty of Medicine and Health Sciences, University of Antwerp, Belgium; Antwerp Unit for Data Analysis and Computation in Immunology and Sequencing (AUDACIS), University of Antwerp, Antwerp, Belgium; Antwerp Center for Translational Immunology and Virology (ACTIV), Antwerp, Belgium; Centre for Health Economics Research and Modeling Infectious Diseases (CHERMID), University of Antwerp, Belgium; Department of Clinical Sciences, Institute of Tropical Medicine, Antwerp, Belgium; Department of General Internal Medicine, Infectious Diseases and Tropical Medicine, University Hospital Antwerp, Belgium; Antwerp Unit for Data Analysis and Computation in Immunology and Sequencing (AUDACIS), University of Antwerp, Antwerp, Belgium; Adrem Data Lab, Department of Computer Science, University of Antwerp, Antwerp, Belgium; Antwerp Center for Translational Immunology and Virology (ACTIV), Antwerp, Belgium; Centre for Health Economics Research and Modeling Infectious Diseases (CHERMID), University of Antwerp, Belgium; Vaccine and Infectious Disease Institute, University of Antwerp, Belgium; Department of Paediatrics, Antwerp University Hospital, Antwerp, Belgium; Antwerp Unit for Data Analysis and Computation in Immunology and Sequencing (AUDACIS), University of Antwerp, Antwerp, Belgium; Adrem Data Lab, Department of Computer Science, University of Antwerp, Antwerp, Belgium; Antwerp Unit for Data Analysis and Computation in Immunology and Sequencing (AUDACIS), University of Antwerp, Antwerp, Belgium; Adrem Data Lab, Department of Computer Science, University of Antwerp, Antwerp, Belgium; Clinical Virology Unit, Department of Clinical Sciences, Institute of Tropical Medicine, Antwerp, Belgium

**Keywords:** immunoinformatics, TCR sequencing, COVID-19, NGS-based diagnostics, T cells, immunology

## Abstract

T-cell–based diagnostic tools identify pathogen exposure but lack differentiation between recent and historical exposures in acute infectious diseases. Here, T-cell receptor (TCR) RNA sequencing was performed on HLA-DR^+^/CD38^+^CD8^+^ T-cell subsets of hospitalized coronavirus disease 2019 (COVID-19) patients (n = 30) and healthy controls (n = 30; 10 of whom had previously been exposed to severe acute respiratory syndrome coronavirus 2 [SARS-CoV-2]). CDR3α and CDR3β TCR regions were clustered separately before epitope specificity annotation using a database of SARS-CoV-2–associated CDR3α and CDR3β sequences corresponding to >1000 SARS-CoV-2 epitopes. The depth of the SARS-CoV-2–associated CDR3α/β sequences differentiated COVID-19 patients from the healthy controls with a receiver operating characteristic area under the curve of 0.84 ± 0.10. Hence, annotating TCR sequences of activated CD8^+^ T cells can be used to diagnose an acute viral infection and discriminate it from historical exposure. In essence, this work presents a new paradigm for applying the T-cell repertoire to accomplish TCR-based diagnostics.

## Teaser

T-cell receptor (TCR) repertoire mining on selected activated T-cell subsets and subsequent epitope annotation successfully identifies SARS-CoV-2–infected individuals and distinguishes them from previously exposed individuals, putting forward a new paradigm of applying the T-cell repertoire to accomplish TCR-based diagnostics.

T cells are part of adaptive immunity and play a crucial role in the cell-mediated immune response against viruses. Antigenic peptides attached to major histocompatibility complex (MHC) molecules of antigen-presenting and infected cells are recognized by the T-cell receptor (TCR). The TCR heterodimer is composed of an alpha (TCRα) and beta (TCRβ) chain in 95% of human T cells (and of a gamma [TCRγ] and delta [TCRδ] chain in the other 5%). During T-cell maturation, each thymocyte develops its own TCR variant by recombination of distinct V, D, and J gene segments, as well as random deletion and/or insertion of nucleotides at junctions. This results in a very broad TCR repertoire, which is essential for enhancing the protective immunity's potential coverage of pathogens and antigens [1]. Recognition of foreign antigenic peptides, presented by MHC, results in T-cell activation and clonal expansion. Theoretically, given enough sequencing power, a skewed repertoire should be detected in T cells directed towards specific antigens in an (infectious) disease context [2, 3]. Hence, the TCR repertoire has the potential to be a diagnostic marker for infections or other diseases involving T-cell responses [4].

Recent advances have been made in leveraging the TCR repertoire to identify previous viral pathogen exposures [5–9]. These methods universally work by identifying disease-associated TCRs that are enriched in patients over controls. This process requires large training cohorts and holds no guarantee that the identified signatures are truly pathogen- or disease-derived. In addition, they detect pathogen exposures without providing information on the timing of this exposure (ie, they do not distinguish historical from recent exposures). The latter is a prerequisite for diagnosing acute infections. Therefore, a TCR-based method that specifically identifies recent exposures (and differentiates these from historical exposures) has yet to be described.

To overcome these limitations, we herein leveraged the activated T-cell status in combination with TCR–epitope annotation to diagnose acute viral infections. Coexpression of the human leukocyte antigen DR (HLA-DR) and CD38 is associated with activation of CD8^+^ T cells in various infections, including severe acute respiratory syndrome coronavirus 2 (SARS-CoV-2) [10–14]. In this study, we demonstrate the diagnostic potential of TCR sequencing in these CD3^+^CD8^+^HLA-DR^+^CD38^+^ T-cell subsets (hereafter called the activated subset) in the context of an acute SARS-CoV-2 infection. Furthermore, we utilize the recent advancements in TCR–epitope annotation to construct a diagnostic framework based on the identification of T-cell reactivity to the target pathogen.

## METHODS

### COVID-19 Patients and Controls

**Figure 1.**
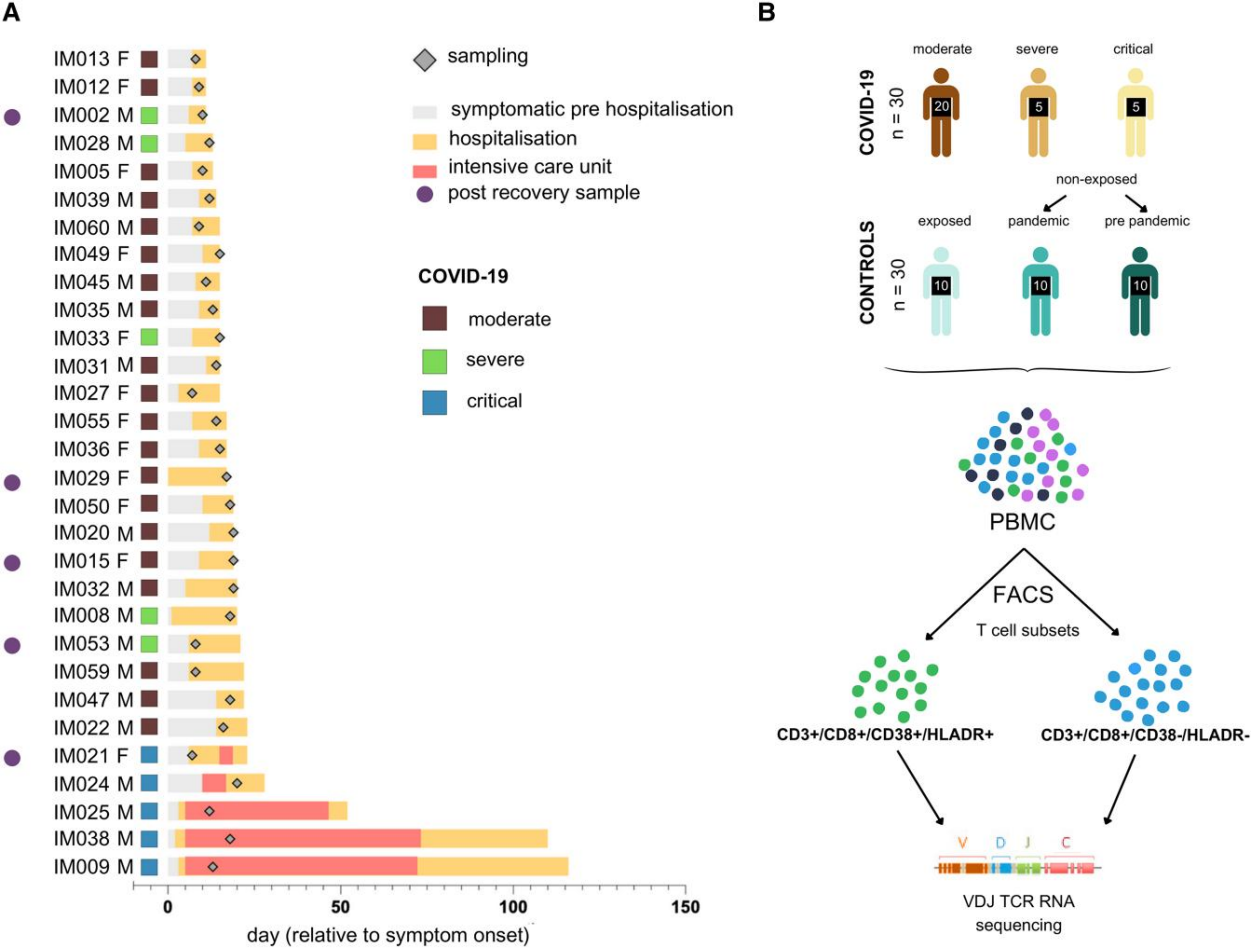
Characterization of sorted T-cell fractions of coronavirus disease 2019 patient and healthy controls. *A*, Patient overview. One patient represents 1 line where severity, sex, symptomatic prehospitalization days, days in the hospital, and time points of sampling are shown. Patients are ordered according to duration of symptomatic period. Of 5 patients, a postrecovery sample was analyzed. *B*. Laboratory method workflow. Study volunteer blood samples were collected from which peripheral blood mononuclear cells were isolated and sorted to obtain T-cell subsets: “activated T cells” (CD3^+^/CD8^+^/CD38^+^/HLA-DR^+^) and “nonactivated T cells” (CD3^+^/CD8^+^/CD38^−^/HLA-DR^–^). RNA from the sorted cells underwent high-throughput T-cell receptor VDJ sequencing. Abbreviations: COVID-19, coronavirus disease 2019; F, female; FACS, fluorescence-activated cell sorting; M, male; PBMC, peripheral blood mononuclear cell; TCR, T-cell receptor; VDJ, variable (V), joining (J), and diversity (D) gene segments.

Recruitment of volunteers for this study (NCT04368143) titled ‘COVID-19 Immune Repertoire Sequencing (IMSEQ)’, was approved by the Antwerp University Hospital ethical committee (reference number 20/12/135) and the Institute of Tropical Medicine Antwerp institutional review board (reference number 1371/20). The Supplementary Methods give a detailed description of the included samples. Figure 1*A* provides a schematic overview of the selected patients with coronavirus disease 2019 (COVID-19) and healthy controls. Supplementary Figure 1 gives an overview of the sampling day and age group of all COVID-19 patient study volunteers. Supplementary Table 1 provides an overview of all the sequenced samples, including WANTAI SARS-CoV-2 antibody enzyme-linked immunosorbent assay results.

### Sample Collection and Fluorescence-Activated Cell Sorting

Figure 1 *B* provides a schematic overview of the wet lab study design. Whole blood samples were obtained from each study participant using lithium heparin tubes. Peripheral blood mononuclear cells (PBMCs) were separated by density gradient centrifugation with Ficoll using standard procedures. The same freezing and thawing procedures were applied to all samples. After thawing, PBMCs were stained using the following directly conjugated antibodies before flow cytometric analysis: anti-human CD3-PerCP (Miltenyi), CD8-APC (Miltenyi), HLA-Dr-V450 (BD Biosciences), and CD38-APC (BioLegend). Data acquisition and cell sorting was performed on a FACSAria II (BD Biosciences). Fractions of 10 000 CD3^+^CD8^+^CD38^+^HLA-DR^+^ and CD3^+^CD8^+^CD38^–^HLA-DR^–^ cells were sorted (as per the gating strategy shown in Supplementary Figure 2) directly into DNA/RNA shield (Zymo Research) and stored at −80°C until further use. Detailed information on the flow-sorted PMBC cell fractions (%CD3^+^ from lymphocytes, %CD8^+^ from CD3^+^ cells, %HLA-DR^+^CD38^+^ from CD3^+^CD8^+^ cells) is shown in Supplementary Table 1.

### RNA Extraction, Library Preparation, and TCR Sequencing

Total RNA was extracted using the Quick RNA microprep kit (Zymo Research) following the manufacturer's protocol and eluted in preheated (65°C) DNAse/RNAse free H_2_O. TCRα, β, γ, and δ chains were amplified using the QIAseq Immune Repertoire RNA Library kit (Qiagen). Following quality control with TapeStation (Agilent), the concentration was determined using the Qubit dsDNA HS Assay kit (Thermo Fisher Scientific), and a pool was made at equimolar amounts of each library. The denatured pooled library (16 pM) was sequenced on the NextSeq platform (Illumina).

### TCR Repertoire Data Analysis

Supplementary Figure 3 provides a schematic overview of the applied analysis methods. In brief, a SARS-CoV-2 TCR database was constructed from publicly available SARS-CoV-2 epitope–TCR pair knowledge. This database was then used to calculate the SARS-CoV-2–associated TCR depth. The Supplementary Methods provide a detailed description of the used SARS-CoV-2-TCR database and applied TCR repertoire analyses, including a detailed definition of SARS-CoV-2 associated depth. The R package Immunarch was used to calculate the Chao diversity metrics and to visualize the dynamics of the TCRs during disease and after recovery [15]. Statistical testing was performed using the open-course statistics package R. Differences were considered statistically significant at a *P* value < .05. To compare the performance of different receiver operating characteristic (ROC) area under the curve (AUC) classifiers, *P* values were calculated as described in [16].

## RESULTS

### Sample Description and Data Acquisition

Basic information of the study volunteers (disease severity, age, sex and day from symptom onset) and the flow-sorted PMBC cell fractions (%CD3^+^ from lymphocytes, %CD8^+^ from CD3^+^ cells, %HLA-DR^+^CD38^+^ from CD3^+^CD8^+^ cells) are summarized in Table 1 and Figure 1. Of the COVID-19 patient group’s (n = 30) CD3^+^ cell fractions, on average, 24.4 ± 11% were CD8^+^ T cells, of which 10.7 ± 7.3% were HLA-DR^+^CD38^+^. In the healthy control group’s (n = 30) CD3^+^ cell fractions, on average, 29.7 ± 10.1% were CD8^+^ T cells, of which 2.9 ± 2.1% were HLA-DR^+^CD38^+^.

**Table 1.** Basic Information of the Study Volunteers and the Flow-Sorted Peripheral Blood Mononuclear Cell Fractions

Characteristic	COVID-19			Controls		
	Moderate (n = 20)	Severe (n = 5)	Critical (n = 5)	Exposed (n = 10)	Nonexposed (n = 10)	Nonexposed Pre-pandemic (n = 10)
Age, y	54.7 ± 9.9	57.8 ± 17.9	60.0 ± 13.8	56.0 ± 10.1	44.3 ± 10.8	28.9 ± 2.6
Sex, No.						
Male	11	4	4	5	5	4
Female	9	1	1	5	5	6
CD3^+^ lymphocytes from PMBC fraction, %	24.0 ± 14.2	55.6 ± 29.3	45.5 ± 28.0	70.1 ± 8.3	71.3 ± 6.9	65.1 ± 7.6
CD8^+^ from CD3^+^ fraction, %	24.0 ± 9.4	33.0 ± 17.0	17.34 ± 4.1	30.9 ± 14.7	27.3 ± 1.6	30.9 ± 1.0
CD38^+^HLA-DR^+^ from CD3^+^/CD8^+^ fraction, %	9.3 ± 5.5	33.0 ± 13.4	10.7 ± 3.9	4.1 ± 2.9	2.48 ± 1.6	2.2 ± 1.0
Days from symptom onset, No.	13.55 ± 4.1	12.6 ± 4.0	14.0 ± 5.1	218 ± 108	NaN	NaN

Abbreviations: COVID-19, coronavirus disease 2019; PBMC, peripheral blood mononuclear cell.

Sequencing was carried out on 10 000 FACS sorted activated CD8^+^ T cells from each donor, as well as on 10 000 FACS sorted nonactivated CD8^+^ T cells from 26 COVID-19 patients and all 10 previously SARS-CoV-2–exposed healthy controls. The number of sequencing reads generated from each donor varied from 1.4 million to 4.5 million, with an average of 2.9 million. Within the COVID-19 acute patient group, the TCR repertoire size (all CDR3α and CDR3β, independent of SARS-CoV-2 specificity) was on average 663 ± 430 (n = 30) for the activated and 1713 ± 795 (n = 26) for the nonactivated CD8^+^ T-cell subsets. In the controls, the TCR repertoire was on average 773 ± 561 (n= 30) for the activated and 1370 ± 575 (n = 10) for the nonactivated CD8^+^ T-cell subsets.

### TCR Repertoires in the Activated CD8^+^ T-Cell Subset Show Features of T-Cell Skewing and Increased Presence of SARS-CoV-2–Associated T-Cell Clones Compared to the Nonactivated CD8^+^ T-Cell Subset in the COVID-19 Patient Group

**Figure 2.**
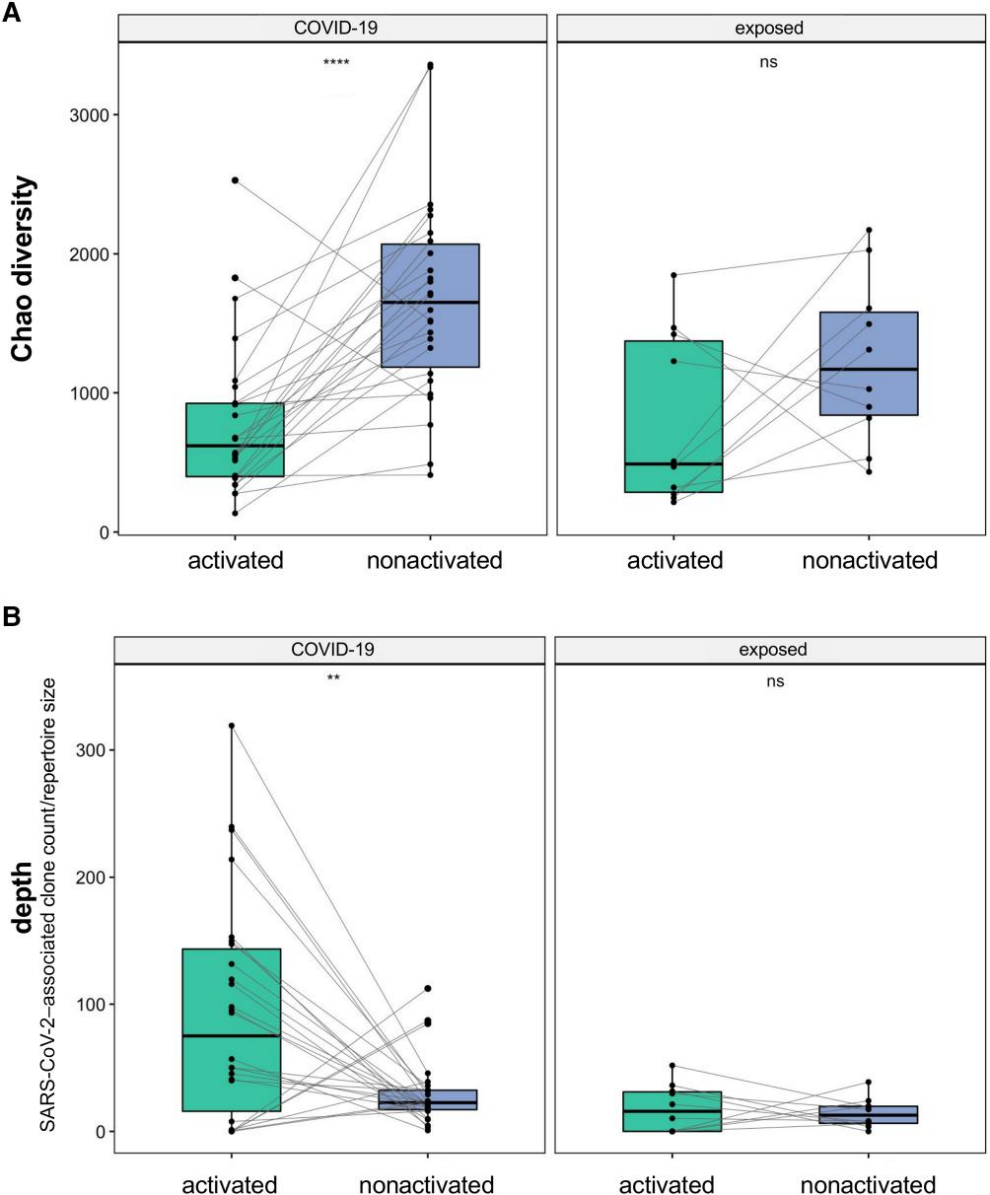
Increased presence of severe acute respiratory syndrome coronavirus 2 (SARS-CoV-2)–associated T-cell receptors (TCRs) in the skewed activated T-cell subset during acute coronavirus disease 2019 (COVID-19). The figure depicts only those patient samples in which both the activated T-cell subset and the nonactivated T-cell subset were analyzed (left: COVID-19 patients, n = 26; right: previously SARS-CoV-2–exposed individuals, n = 10) *A*, Chao1 diversity (sum of Chao1 index of the CDR3α and CDR3β chains). *B*, Clonal expansion of SARS-CoV-2–associated T cells expressed as SARS-CoV-2–associated TCR depth (defined as the sum of SARS-CoV-2–associated TCR clones divided by the repertoire size). *P* values denote paired Student *t* test. **P ≤ .01; ****P ≤ .0001; ns, not significant (*P* > .05).

The Chao diversity index, a measure of TCR repertoire diversity [1, 17–19], demonstrates that the activated CD8^+^ T-cell subset contains a less diverse repertoire than the nonactivated CD8^+^ T-cell subset. This was statistically significant within the COVID-19 patient group (n = 26), but not in the previously exposed healthy control group (n = 10) (Figure 2*A*). This decreased diversity is indicative of a skewed TCR repertoire driven by clonal expansion. Then we analyzed whether the skewed TCR repertoire in the activated CD8^+^ T-cell subset in the COVID-19 patient group was associated with an increased presence of SARS-CoV-2–associated T-cell clones. Indeed, the SARS-CoV-2–associated TCR depth was significantly higher in the activated CD8^+^ T-cell subset than in the nonactivated CD8^+^ T-cell subset of the same individuals within the COVID-19 patient group, but not in the previously exposed healthy control group (Figure 2*B*). The increase in SARS-CoV-2–associated TCR depth in COVID-19 patients compared to previously exposed individuals is more pronounced in the activated CD8^+^ T-cell subset (fold increase = 5) compared to the increase observed in the nonactivated CD8^+^ T-cell subset (fold increase = 1.5) (Supplementary Figure 4). This underscores our hypothesis that the activated CD8^+^ T-cell subset, more than the nonactivated CD8^+^ T-cell subset, allows discrimination between patient and control groups (even in the context of historical exposure to the pathogen).

### Within-Patient Tracking Shows Depletion of SARS-CoV-2–Associated TCRs in the Activated CD8^+^ T-Cell Subsets Upon Recovery From Infection

**Figure 3.**
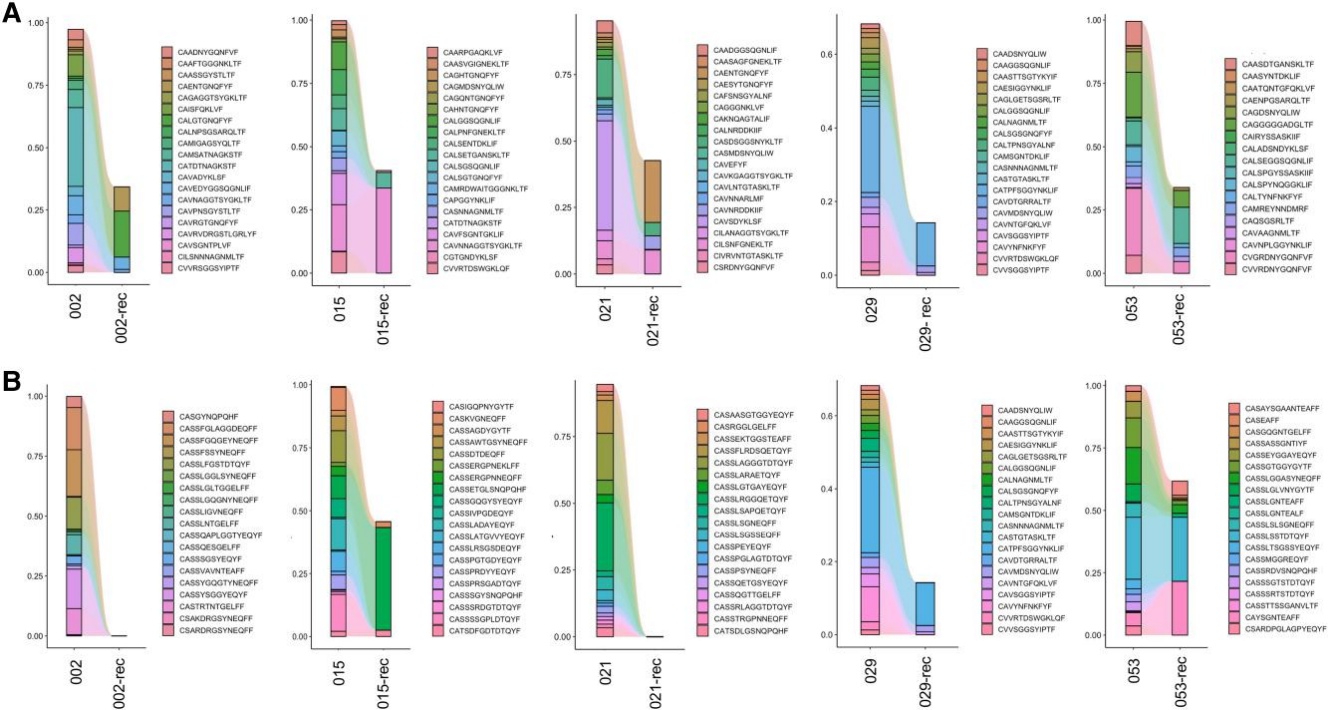
Severe acute respiratory syndrome coronavirus 2 (SARS-CoV-2)–associated clonotype frequency in CD8^+^/CD38^+^/HLA-DR^+^ populations detected during active disease and after recovery (rec) in the same individuals (n = 5). *A*, SARS-CoV-2 CDR3α matches. *B*, SARS-CoV-2 CDR3β matches. Depicted are the top 20 SARS-CoV-2–associated T-cell receptors after coronavirus disease 2019 (COVID-19) recovery compared to the acute COVID-19 phase.

Five of the included COVID-19 patients have been followed longitudinally; hence, paired samples were available spanning the acute infection and recovery phases. In the activated CD8^+^ T-cell subset of these 5 individuals, we observed a depletion of the SARS-CoV-2–associated clonotype frequency in the convalescent phase (after recovery) compared to the acute infection phase (Figure 3). This was consistent for both the α- and β-chain matches. This observation once more verifies (on an individual patient resolution) that after recovery, a depletion in pathogen-associated TCRs occurs in the activated CD8^+^ T-cell subset. This further highlights the potential of infection-associated TCRs within activated CD8^+^ T-cell subsets to serve as a diagnostic marker for acute viral infections and differentiation with historical exposures to the same pathogen.

### Depth of the SARS-CoV-2–Associated Activated CD8^+^ T-Cell Subset Differentiates COVID-19 Patients From Healthy Controls

**Figure 4.**
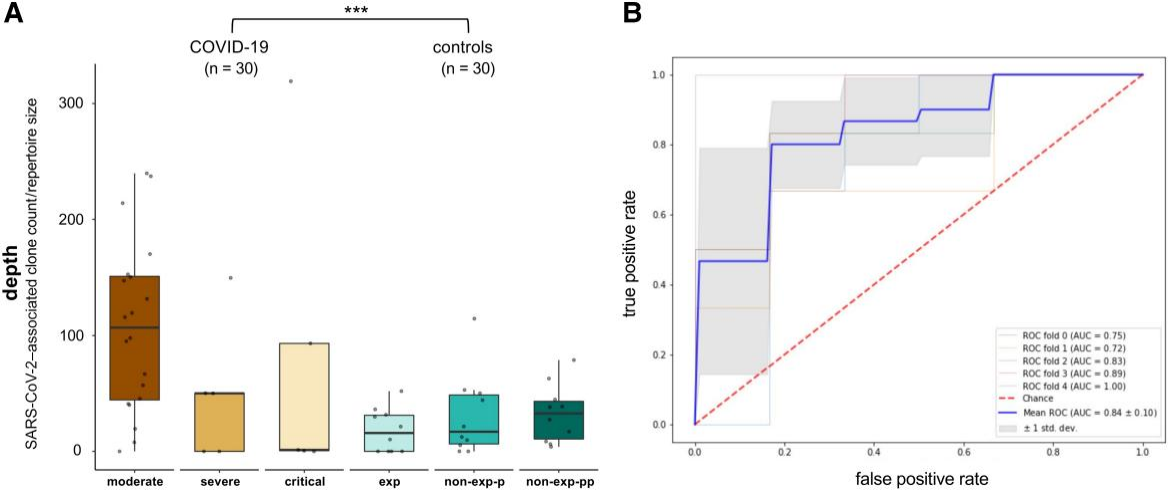
Severe acute respiratory syndrome coronavirus 2 (SARS-CoV-2) predictive performance of CD8^+^/CD38^+^/HLA-DR^+^ populations. *A*, Depth of the SARS-CoV-2–associated immune response. Of the 30 included patients, 20 had moderate, 5 severe, and 5 critical coronavirus disease 2019. In the control group, 10 individuals had past SARS-CoV-2 infection (exposed [exp]), 10 had no evidence of a previous SARS-CoV-2 infection although being sampled during the pandemic (nonexposed-pandemic [non-exp-p]), and 10 were non-exposed controls sampled before the pandemic (nonexposed-prepandemic [non-exp-pp]). *P* value denotes Student *t* test. ***P ≤ .001. *B*, Receiver operating characteristic curve using the SARS-CoV-2–associated depth in a logistic regression classifier. Abbreviations: AUC, area under the curve; COVID-19, coronavirus disease 2019; ROC, receiver operating characteristic; SARS-CoV-2, severe acute respiratory syndrome coronavirus 2.

After establishing that the skewed TCR repertoire observed in the activated CD8^+^ T-cell subset (compared to the nonactivated subset) is linked with a significant enrichment of SARS-CoV-2–associated TCRs in COVID-19 patients, we then validated that enumeration of SARS-CoV-2–associated TCRs in this subset is a diagnostic marker for COVID-19. The SARS-CoV-2–associated depth differed significantly between the COVID-19 patients (n = 30) and all healthy controls (n = 30) (Figure 4*A*) (*P* = .0002). As an additional control, the influenza-associated depth did not differ between the COVID-19 patients and healthy controls (Supplementary Figure 5). A logistic regression classifier using SARS-CoV-2–associated TCR depth of activated CD8^+^ T cells in COVID-19 patients versus all healthy controls generated a ROC AUC of 0.84 ± 0.10 after 5-fold cross-validation (Figure 4*B*). While our method seems more performant when using the CDR3α and CDR3β chain information combined (compared to either the CDR3α or CDR3β chain information alone), and in samples taken day 14–21 versus day 7–14 after symptom onset, these effects were not statistically significant (*P* = .3, *P* = .1, and *P* = .19, respectively) (Supplementary Figure 6*A*, 6*B*, 6*E*, 6*F*). We did observe a significantly increased performance in cases with moderate compared to severe and critical COVID-19 disease (*P* = .0014) (Supplementary Figure 6*C* and 6*D*). Cytomegalovirus (CMV) serostatus did not influence the SARS-CoV-2–associated ROC (Supplementary Figure 6*G* and 6*H*) (*P* = .35). Finally, putative common-cold cross-reactive TCRs did not significantly contribute to the SARS-CoV-2–associated TCR depth since database depletion of these TCRs did not affect the performance of the classifier (*P* = .5) (Supplementary Figure 7).

## DISCUSSION

Infectious disease laboratory diagnosis uses technologies that can detect either pathogen-derived material (antigenic or genomic) directly or indirectly by their immunological imprint. The latter is particularly important in infectious diseases where direct detection is challenged (eg, by low or short pathogenic shedding). T cells, just like B cells (and derived antibodies), are a crucial component of the immune system, playing a central role in the detection and elimination of infected and malignant cells. Moreover, T cells are involved in activation of the humoral response and thus potentially precede detectable antibody signals. Indirect diagnostic assay designs, however, remain predominated by antibody detection (ie, serologic testing). Interestingly, T cells persist over time, sometimes even in the absence of seroconversion [20, 21]. Accordingly, some individuals do not generate detectable antibody responses [22]. Improved sensitivity of T-cell–based tests over antibody testing has been asserted for detection of SARS-CoV-2 exposure [9, 23] and prediction of COVID-19 protection [24]. Despite potential advantages over antibody detection, T-cell–based assays remain scarce in the laboratory diagnostic landscape. Beyond logistical reasons, the inability of current T-cell assays to differentiate recent from past pathogen exposures constrains their diagnostic utility. In some instances, however, the mere identification of pathogen exposure suffices to provide clinically relevant diagnostic information. In the control of *Mycobacterium tuberculosis* (TB), the identification of both patients with active disease and latent TB infection is important [25]. In latent TB, demonstration of historical pathogen exposure is achieved with T-cell–based diagnostic techniques (eg, QuantiFERON-Gold, Qiagen; TB-IGRA, Wantai; T-SPOT.TB, Oxford Immunotec). Of note, the TB T-cell assays cannot be used to distinguish latent from active TB.

Contrary to diagnosing chronic or latent infectious diseases, acute infectious disease diagnosis relies on distilling recent pathogen exposure information. In such a case, a general image of pathogen exposures (ie, a combination of recent and historical exposures) will not suffice—or even hamper—to explain a patient's clinical presentation. To derive information on the timing of pathogen exposure, serological assays exploit immunoglobulin class switching (primarily the immunoglobulin M to immunoglobulin G isotype switch) induced by B-cell activation during in vivo processes following antigen recognition. Likewise, information on T-cell markers associated with T-cell activation could broaden the clinical spectrum of T-cell–based laboratory assays. Traditional T-cell assays, however, consist of measuring cytokines released by ex vivo antigen-stimulated T cells (such as the enzyme-linked immunosorbent spot assay and the interferon-γ release assay). As such, the original in vivo activation status of T cells recognizing pathogen-derived antigens is not registered.

The in vivo T-cell activation process is driven by the recognition of antigenic peptides by the TCR. Recently developed TCR repertoire profiling technology (through TCR sequencing) allows identification of infectious disease–associated T-cell imprints. For instance, an exposure classifier based on the TCR repertoire distinguished naive from smallpox-vaccinated mice [8], Zika virus–exposed from dengue virus–exposed mice [7], and CMV-seropositive from CMV-seronegative human individuals [5, 6]. Recently, the first test based on this technology received US Food and Drug Administration approval for the detection of SARS-CoV-2 exposure (T-Detect, Adaptive Biotechnologies [9]). Just as with traditional T-cell assays, however, this TCR profiling technology merely indicates pathogen exposure.

The basis of our diagnostic classifier involves the annotation of TCR sequences with their cognate epitopes. This is in large contrast with these prior attempts to create TCR-based disease classifiers, which have searched for enriched TCRs within patient cohorts [5, 9]. That method requires very large training patient cohorts, and the identified disease-associated TCRs do not necessarily have their target epitope verified. With this study, we put forward a new paradigm in the way T-cell repertoire data can be applied to accomplish TCR-based diagnostics. In contrast to enrichment-based methods, smaller patient cohorts can be applied as most of the training information is derived from the epitope–TCR databases.

By using the TCR profiling technology in conjunction with markers of T-cell activation, we demonstrate a T-cell–based assay that differentiates recent from historical pathogen exposure. This advance could pave the way for a broader introduction of T-cell information in a diagnostic setting. Since extensive information about SARS-CoV-2 epitope-specific TCRs has become available, we focused on COVID-19 to deliver the first proof of such a concept. Using TCR sequencing of 10 000 CD8 T cells expressing activation markers (CD38 and HLA-DR) sampled from each of the 30 COVID-19 patients and 30 healthy controls, the SARS-CoV-2 infection status was inferred by annotating those TCRs that are expected to react to a SARS-CoV-2 epitope (ROC AUC of 0.84 ± 0.10). Among the COVID-19 group, samples were taken ranging from day 7 to day 21 post–symptom onset and from patients with different disease severities (moderate, severe, and critical). In accordance with the observation that the CD8^+^ T-cell signal peaks 14 days after COVID-19 symptom onset [26], our method seemed more performant on samples taken later after symptom onset (days 14–21 compared to days 7–14), although this effect was not statistically significant. In addition, our diagnostic classifier underperformed in COVID-19 patients with the most severe disease course. This could be due to T-cell depletion or delayed T-cell responses in more severe COVID-19 cases [26–28]. Evidence for T-cell apoptosis in severe COVID-19 has been described previously [27]. While overall T-cell counts do not differ between our moderate, critical, and severe COVID-19 patients (Supplementary Figure 8), SARS-CoV-2–specific T-cell counts might differ. Of note, our study recruitment strategy did not allow us to investigate our classifier in very mild and asymptomatic individuals. It is interesting to note here that compared to CD8 T cells, CD4 T cells are more consistently observed (which could further improve SARS-CoV-2–associated TCR signal consistency) in patients with different disease severities than CD8 T cells [29–31]. While past CMV exposure has been shown to impact the TCR repertoire [32], CMV serostatus did not influence our classifier. Of note, since underlying active coinfections (like human immunodeficiency virus, hepatitis C virus, and hepatitis B virus) were excluded from the study, we cannot exclude their potential impact on our classifier. While the CDR3β region has been explored most in TCR-based exposure classifiers [6, 7, 9, 33, 34], we observed that the combination of both the CDR3α and CDR3β regions of the TCR tended towards greater diagnostic performance than either of the regions separately (however, no statistical significance was reached).

Beyond specific identification of COVID-19 patients in their acute but not convalescent phase, our T-cell–based assay employs hallmarks of the T-cell response that improve its sensitivity as a diagnostic target as well. The cellular immune response is antigen-specific and is amplified through clonal T-cell expansion. Accordingly, the T-cell subset expressing activation markers (CD38 and HLA-DR), more than the nonactivated subset, allowed appreciation of these T-cell features in our COVID-19 patient group with a significantly increased clonality (reciprocal of diversity) and SARS-CoV-2–associated repertoire size. For most samples, we were able to detect a stronger SARS-CoV-2–associated T-cell response in the activated T-cell subset compared to the nonactivated T-cell subset. For 3 individuals who showed a relatively high SARS-CoV-2–associated T-cell response in the nonactivated subset, it is possible that either alternative T-cell activation markers would be more suitable or that CD8^+^ T-cell activation was compromised [35].

Individual CDR3α and/or CDR3β regions can recognize epitopes from different pathogens. Such cross-reactive TCRs have been involved in response to SARS-CoV-2 infection and are thus part of the SARS-CoV-2 epitope–TCR database [36, 37]. Interestingly, TCR repertoire profiling was shown to differentiate exposure with similar viruses (Zika virus from dengue virus [7]). Since our study population does not include patients infected with co-circulating viruses related to SARS-CoV-2 such as seasonal coronaviruses, we cannot demonstrate whether our classifier differentiates acute SARS-CoV-2 from acute seasonal coronavirus infection. Of note, most of our participants must have experienced seasonal coronavirus exposures in the past [38]. Interestingly, depletion of our SARS-CoV-2 epitope–TCR database with TCRs identified as putative common-cold coronaviruses (human coronaviruses OC43, HKU1, 229E, and NL63) cross-reactive did not affect the performance of our classifier.

Some opportunities for future development exist. While we assembled a SARS-CoV-2–associated TCR database, epitope–TCR information is available for many more infectious diseases. Our technology, therefore, has the potential to identify recent exposures to different pathogens or finally even allow for agnostic diagnostic testing (ie, without needing a predefined diagnostic target), hence abolishing the need for multiple parallel analyses on an individual patient sample. Just as with the recent explosion of SARS-CoV-2 TCR knowledge, we expect our method to continuously improve with ever-growing publicly available epitope–TCR data. Furthermore, as epitope–TCR annotation methods become more accurate, so too will any derived diagnostic classifier. Additionally, the emergence of single-cell TCR sequencing might further improve specificity due to the mining of paired α- and β-chain information belonging to an individual T-cell receptor (as both chains are involved in antigen recognition) [39]. It will also be important for TCR databases to well-represent different HLA types. Of note, we did not determine or select patients based on ethnic background or predefined HLA types. To confirm the HLA-independent performance, additional studies should focus on including a broad representation of ethnic backgrounds.

In conclusion, presently available T-cell–based technologies are not designed to differentiate recent from historical pathogen exposures. We demonstrate that our approach based on TCR repertoire mining on selected activated T-cell subsets and subsequent epitope annotation successfully identifies SARS-CoV-2–infected individuals and distinguishes them from previously exposed individuals. This innovation will boost the implementation of T-cell–based information in a broader (infectious disease) diagnostic context.

## Supplementary Data

Supplementary materials are available at *The Journal of Infectious Diseases* online. Consisting of data provided by the authors to benefit the reader, the posted materials are not copyedited and are the sole responsibility of the authors, so questions or comments should be addressed to the corresponding author.

## Supplementary Material

jiad430_Supplementary_Data

## Notes

***Acknowledgments***. We express our sincere gratitude to all individuals who participated in the IMSEQ study. Their willingness and cooperation were essential for the successful completion of this research. Special thanks are extended to Marianne Mangelschots, Charlotte Drieghe, and Sergio Garcia for their exceptional dedication in the laboratory. Additionally, we acknowledge the Priorix vaccination study volunteers whose samples were included in the nonexposed prepandemic group. Finally, we extend our appreciation to all the researchers, clinicians, and staff members who contributed to the data collection and analysis. Their contributions are greatly appreciated.

***Author contributions***. K. V., P. M., K. L., B. O., and A. V. conceptualized the study. A. V., K. V., P. M., K. L., B. O., E. B., G. E., W. A., G. V., P. V. D., V. V. T., P. B., E. V., and G. O. designed the study methodology. L. v. P., I. B., C. T., S. v. I., M. v. F., and E. V. were involved in clinical study protocol revision, implementation, patient recruitment, management, and clinical data collection. A. V., M. H., T. d. B., and E. B. conducted experiments. A. V. and K. V. wrote the original draft of the manuscript. P. M., K. L., B. O., W. A., G. V., and S. v. I. edited the draft manuscript. All authors reviewed the final manuscript version.

***Data availability***. The authors declare that all data supporting the findings of this study are available from the corresponding author upon request.

***Disclaimer***. ImmuneWatch had no role in study design, data collection and analysis, decision to publish, or preparation of the manuscript.

***Financial support***. This work was supported by the Flemish Government Science and Innovation (Departement Economie, Wetenschap en Innovatie (EWI): Joint Pump Priming Project to K. V., W. A., B. O., K. L., and P. M.); the Research Foundation Flanders (1861219N to B. O.); the Hercules Foundation Flanders; University of Antwerp concerted research action (to P. B., V. V. T., and P. V. D.); University of Antwerp Methusalem VAXINFECTIO funding (to P. B. and P. V. D.); University of Antwerp Excellence Centre ASCID (to P. B. and P. V. D.); and University of Antwerp Excellence Centre MST (to K. L.).
