# Supplementary material for: Diagnosing Viral Infections Through T-Cell Receptor Sequencing of Activated CD8+ T Cells
Source: J Infect Dis. 2023 Oct 3;229(2):507–16. doi: 10.1093/infdis/jiad430 (PMC10873181; doi:10.1093/infdis/jiad430)
Supplement: jiad430_Supplementary_Data [file jiad430_supplementary_data.pdf]

Diagnosing viral infections through T cell receptor sequencing of activated CD8+ T cells

Alexandra Vujkovic[1,2,3], My Ha[2,4,5,6] , Tessa de Block[7], Lida van Petersen[7], Isabel Brosius[7], Caroline Theunissen[7], Sabrina H. van Ierssel[8], Esther Bartholomeus[2,4], Wim Adriaensen[9], Guido Vanham[10], George Elias[11], Pierre Van Damme[6], Viggo Van Tendeloo[11], Philippe Beutels[2,4,5], Maartje van Frankenhuijsen[7], Erika Vlieghe[8], Benson Ogunjimi[2,3,4,5,6,12], Kris Laukens[2,3], Pieter Meysman[2,3,a], Koen Vercauteren[1,a,#]

[1] Clinical Virology Unit, Department of Clinical Sciences, Institute of Tropical Medicine, Antwerp, Belgium

[2] Antwerp Unit for Data Analysis and Computation in Immunology and Sequencing (AUDACIS), University of Antwerp, Antwerp, Belgium

[3] Adrem Data Lab, Department of Computer Science, University of Antwerp, Antwerp, Belgium

[4] Antwerp Center for Translational Immunology and Virology (ACTIV), Antwerp, Belgium

[5] Centre for Health Economics Research and Modeling Infectious Diseases (CHERMID), University of Antwerp, Belgium

[6] Vaccine and Infectious Disease Institute, University of Antwerp, Belgium

[7] Department of Clinical Sciences, Institute of Tropical Medicine, Antwerp, Belgium

[8] Department of General Internal Medicine, Infectious Diseases and Tropical Medicine, University Hospital Antwerp, Belgium

[9] Clinical Immunology Unit, Department of Clinical Sciences, Institute of Tropical Medicine, Antwerp, Belgium

[10] Biomedical Department, Institute of Tropical Medicine, Antwerp, Belgium

[11] Laboratory of Experimental Hematology, Faculty of Medicine and Health Sciences, University of Antwerp, Belgium

[12] Department of Paediatrics, Antwerp University Hospital, Antwerp, Belgium

[a] P. M. and K. V. contributed equally to this work.

[#] corresponding author: Prof. Dr. Koen Vercauteren, +32032476332, Kronenburgstraat 43, 2000 Antwerpen, Belgium, kvercauteren@itg.be

Short title: TCR sequencing of activated CD8+ T cells

Summary: TCR repertoire mining on selected activated T cell subsets and subsequent epitope annotation successfully identifies SARS-CoV-2 infected individuals and distinguishes them from previously exposed individuals, putting forward a new paradigm of applying the T cell repertoire to accomplish TCR-based diagnostics.

A. Supplementary methods

1. COVID-19 patients and controls (detailed description)

COVID-19 patient group (n=30):

Adult (18+, with no age limit) COVID-19 patients admitted to the Antwerp University Hospital with PCR-confirmed SARS-CoV-2 infection were included in this study (April 2020 – November 2021) (NCT04368143). The WHO standards for adults were used to determine COVID-19 disease severity (supp. table 2). At study analysis initiation, a total of 58 patients with varying degrees of COVID-19 severity, encompassing asymptomatic (n=2), mild (n=1), moderate (n=37), severe (n=5), and critical (n=13) cases, had been included. For TCR analysis, a selection of 30 patients was made. We excluding patients with cancer and individuals who passed away during the study period (n = 11). From the remaining 47, 30 patients were included to represent the moderate, severe, and critical severity categories with at least 5 patients included per category and applying an overall balanced distribution in terms of sampling day after symptom onset (day 7-14 after symptom onset (n = 15) and day 14-20 days after symptom onset (n = 15)). Supp. figure 1 provides an overview of the sampling day and age group of all COVID-19 patient study volunteers.

Previously exposed group (n=10):

Healthy volunteers with previous SARS-CoV-2 exposure were recruited at the Institute of Tropical Medicine in Antwerp, Belgium, during the pandemic (first trimester of 2021, before national SARS-CoV-2 vaccination campaigns were initiated). The exclusion criteria were having chronic infectious conditions or having received any vaccinations in the previous two months. The exposed individuals either had a positive serological test result on the day of sampling or a positive SARS-CoV-2 PCR result more than two months before the sample was obtained. Five of them were longitudinally sampled volunteers from the COVID-19 patient group (allowing intra-patient TCR tracking from the acute until the recovery period).

Non-exposed pandemic group (n=10):

Healthy volunteers without evidence of previous SARS-CoV-2 exposure (infection or vaccination) were recruited as well (in parallel with the previously exposed group). These individuals (A) were

unaware of high-risk contacts with positive SARS-CoV-2 cases; (B) reported not having experienced an episode of symptoms that could be linked to a potential SARS-CoV-2 infection since the start of the SARS-CoV-2 outbreak; or (C) had a negative PCR test after a high-risk contact and/or episode of symptoms since the start of the SARS-CoV-2 outbreak. All individuals included in this control group had negative SARS-CoV-2 serologic testing (*WANTAI* SARS-CoV-2 Ab ELISA) on day of inclusion (serology results of all participants are shown in sup table 1).

Non-exposed pre-pandemic group (n=10):

Additionally, samples taken from ten volunteers from a Priorix ® (mumps, measles, and rubella) vaccination study held before the pandemic (thus prior to 2020) were included. This group therefore had a recent immune stimulus other than SARS-CoV-2 exposure (via Priorix ® vaccination)[39].

2. SARS-CoV-2-TCR database construction from publicly available SARS-CoV-2 epitope-TCR pair knowledge

A library of SARS-CoV-2-epitope-TCR pairs was established by combining four primary sources (all accessed on 20/04/2022): (A) VDJdb database (https://vdjdb.cdr3.net/), (B) IEDB database (https://www.iedb.org), (C) the ImmuneCODE collection from Adaptive Technologies and Microsoft, which contained pairs derived through a MIRA assay[39] and (D) single cell TCR data[40]. After duplicate removal, a total of 148,888 publicly available SARS-CoV-2 associated TCRs, corresponding to 1205 epitopes remained. The database was split up into TCRα (i.e., CDR3α) and TCRβ (i.e., CDR3β) databases that were used for downstream analyses.

As a negative control, publicly available influenza epitope TCR repertoire data was collected from the VDJdb and IEDB (total CDR3α: 5402, CDR3β: 8514).

Putative common cold cross-reactive TCRs within our SARS-CoV-2 epitope-TCR database were identified by running NCBI BLAST[41] (version 2.13.0+) on the 1205 SARS-CoV-2 epitopes against four RefSeq genomes of human coronavirus OC43 (HCoV-OC43) (GCF_003972325.1), human coronavirus HKu1 (HCoV-HKU1) (GCF_000858765.1), human coronavirus 229E (HCoV-229E) (GCF_001500975.1) and human coronavirus NL63 (HCoV-NL63) (GCF_000853865.1). One

mismatch between epitopes was allowed. A total of 52 epitopes were found to be shared with at least one of the four common cold coronaviruses, corresponding to 703 CDR3α and 18407 CDR3β sequences. The latter were defined as putative common cold cross-reactive TCRs.

3. TCR repertoire data analysis

The SARS-CoV-2 associated TCR depth was calculated as schematically shown in supp. figure 3. MiXCR was used with default settings for RNA-seq read alignment and assembly, as well as the exporting of clonotypes[42]. The output was split up into alpha chain (CDR3α) and beta chain (CDR3β) clonotypes. To identify clusters of similar CDR3 sequences that in theory recognize the same peptide, ClusTCR[43] was used with default settings on the CDR3α and CDR3β information separately. To identify clusters matching the epitope associated CDR3α and CDR3β from the constructed databases, TCRMatch with a 0.97 cut-off was used[44]. All clusters consisting of minimally three CDR3s were retained. Clusters were labeled SARS-CoV-2 associated in case one of the CDR3s matched the SARS-CoV-2 database (see method section 4.).

We used ‘depth’ to quantitatively express the SARS-CoV-2 associated TCR repertoire, i.e., ‘SARS-CoV-2 associated clone count’ divided by the ‘repertoire size’ using the combination of both CDR3α and CDR3β chains, unless mentioned otherwise. The ‘SARS-CoV-2 associated clone count’ is defined as the total number of CDR3 transcripts belonging to SARS-CoV-2 associated clusters.

The ‘repertoire size’ is defined as the sum of unique clones (i.e., CDR3s with a unique combination of the V-gene, J-gene, and CDR3) in the entire TCR repertoire.

To infer the infection state, the SARS-CoV-2 associated depth was used in Logistic regression by the sklearn.linear model.LogisticRegression function from scikit-learn v1.0.2 in Python 3.9.7[45]. The resulting ROC AUC values were statistically compared as described[46].

To calculate the Chao diversity metrics and to visualize the dynamics of the TCRs during disease and after recovery, the R package Immunarch was used[47].

B. Supplementary figures and tables

Supplementary figure 1: Number of included COVID-19 patient samples per day relative to symptom onset (**A**) and per age group (**B**).

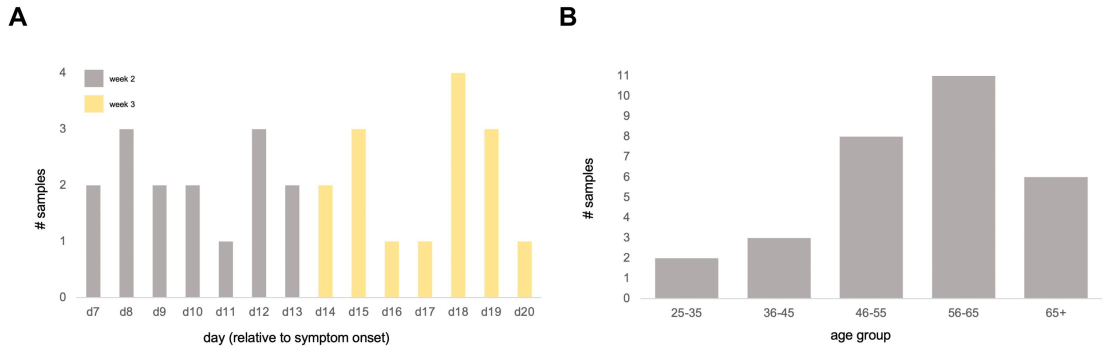

Supplementary figure 2: Flowcytometry gating strategy

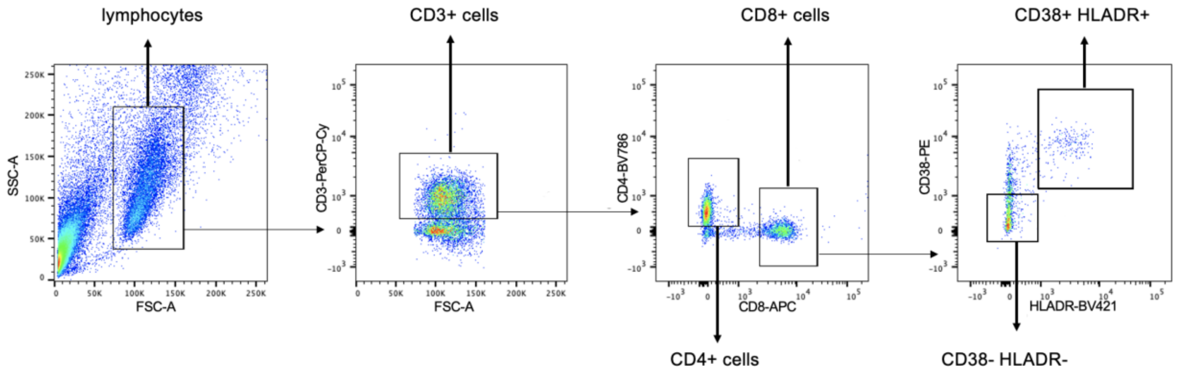

Supplementary figure 3: Analysis flowchart. All TCR sequencing reads were annotated using MiXCR (v3.0.7) (1). The resulting CDR3α and CDR3β sequences were clustered with the ClusTCR tool (2), the resulting clusters were then matched (3) to a database of >140.000 known SARS-CoV-2 associated CDR3α and CDR3β sequences, respectively. TCR cluster database matches were considered SARS-CoV-2 associated.

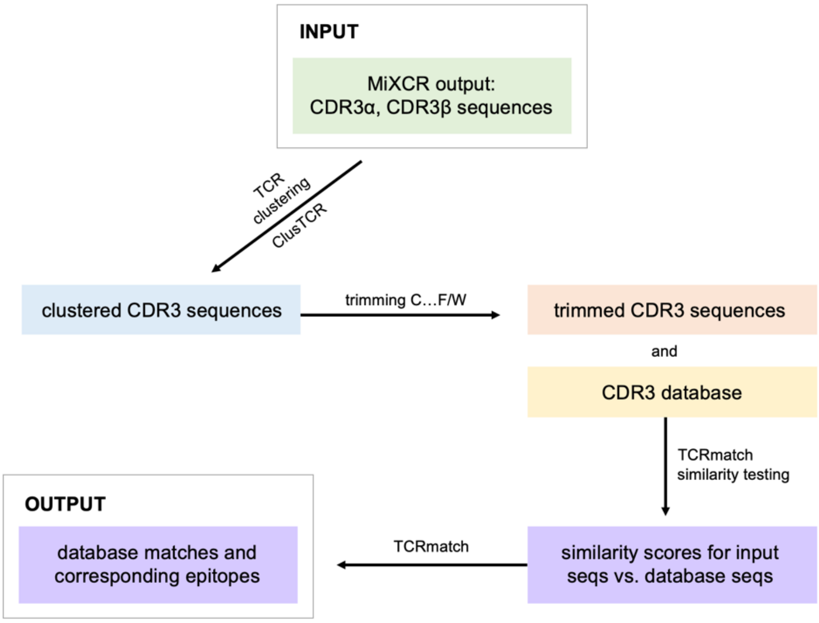

Supplementary figure 4: The increase in SARS-CoV-2 associated TCR depth in COVID-19 patients compared to previously exposed individuals is more pronounced in the activated than the non-activated CD8+ T cell subset. The panel depicts only those patient samples in which both the active T cell subset (green boxes) and the non-active T cell subset (purple boxes) were analysed (COVID-19 patient n = 26 and previously SARS-CoV-2 exposed individuals n = 10). Clonal expansion of SARS-CoV-2 associated T-cells is expressed as SARS-CoV-2 associated TCR depth (defined as the sum of SARS-CoV-2 associated TCR clones divided by the repertoire size). P values denote student T-testing (* p ≤ 0.05, *** p ≤ 0.001).

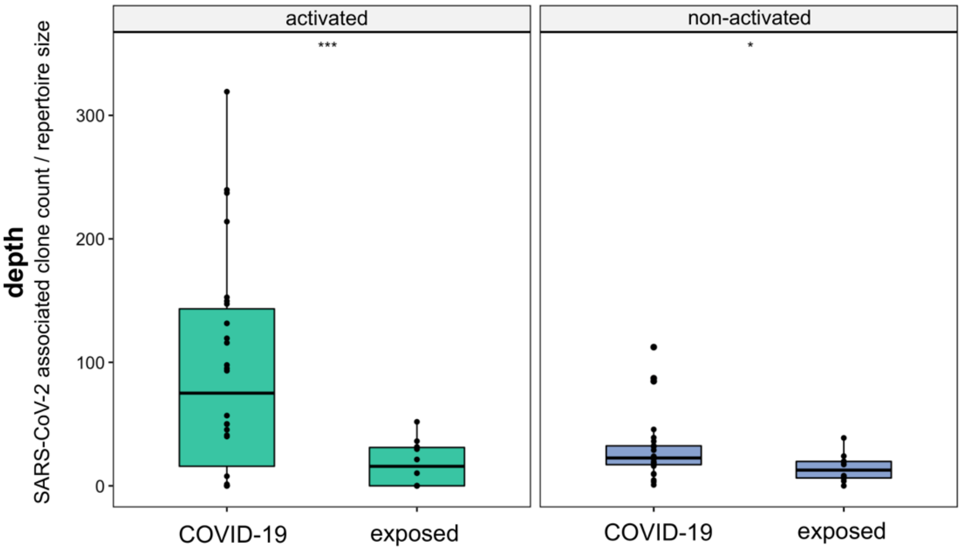

Supplementary figure 5: Influenza associated CD8+/CD38+/HLADR+ TCRs. A. The depth of the influenza associated immune response. Of the thirty included patients, twenty had moderate, five severe, and five critical COVID-19. In the control group, ten individuals had past SARS-CoV-2 infection (exposed (exp)), ten had no evidence of a previous SARS-CoV-2 infection although being sampled during the pandemic (non-exposed-pandemic (non-exp-p)) and ten were non exposed controls sampled before the pandemic (non-exposed-prepandemic (non-exp-pp)). P value denotes student T-testing (non-significant (ns) p > 0.05).

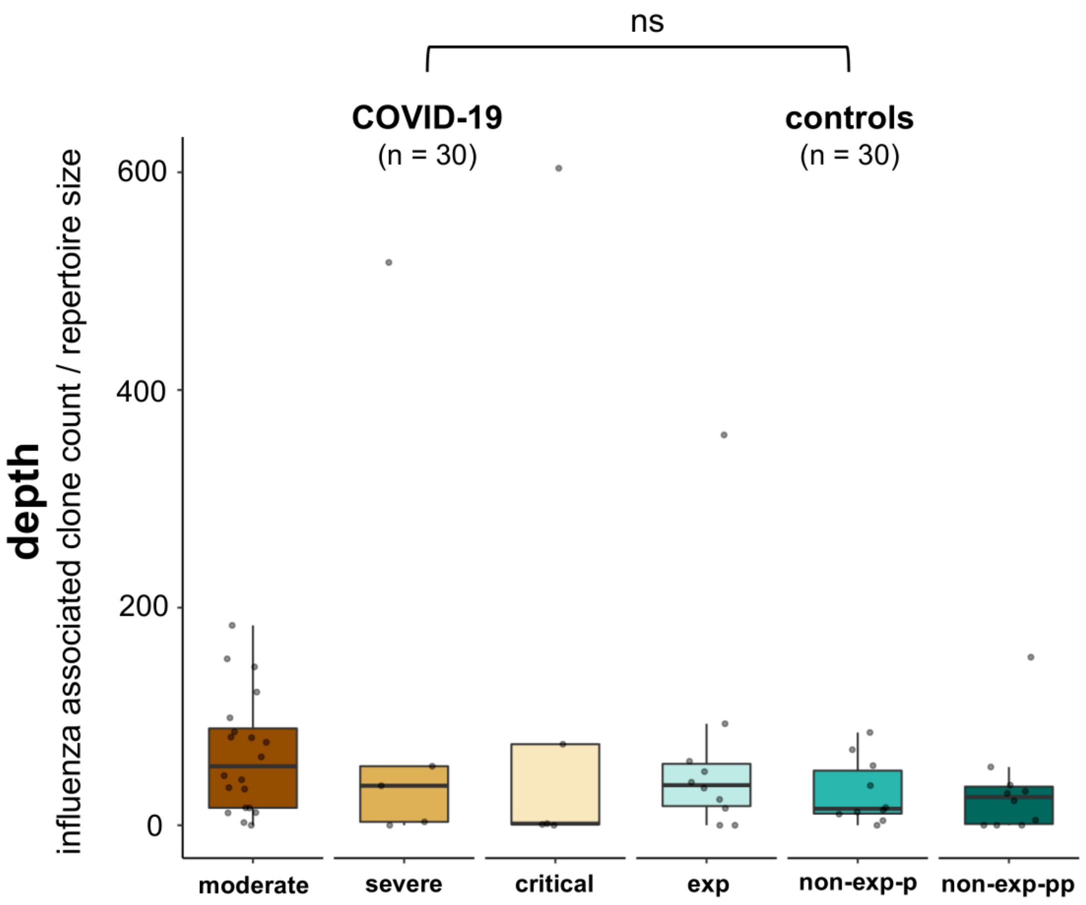

Supplementary figure 6: comparisons of ROC AUCs using the SARS-CoV-2 associated depth in a logistic regression classifier. P values are calculated as described in (23). **A)** ROC analysis of CDR3α sequence database matching. **B)** ROC analysis of CDR3β sequence database matching. (ROC AUC A vs. B p-value = 0.24) **C)** ROC analysis of CDR3α and CDR3β sequence database matching on the patients sampled on day 7-14 from symptom onset (n=15) vs. controls (n=30). **D)** ROC analysis of CDR3α and CDR3β sequence database matching on the patients sampled on day 14-21 from symptom onset (n=15) vs. controls (n=30). (ROC AUC C vs. D p-value = 0.19) **E)** ROC analysis of CDR3α and CDR3β sequence database matching on the patients with moderate COVID-19 (n=20) vs. controls (n=30). **F)** ROC analysis of CDR3α and CDR3β sequence database matching on patients with severe/critical disease (n=10) vs. controls (n=30). (ROC AUC E vs. F p-value = 0.0014) **G)** ROC analysis of CMV seropositive COVID-19 patients (n=18) against CMV seropositive controls (n=14). **H)** ROC analysis of CMV seronegative COVID-19 patients (n=12) against CMV seronegative controls (n=6). (ROC AUC G vs. H p-value = 0.35).

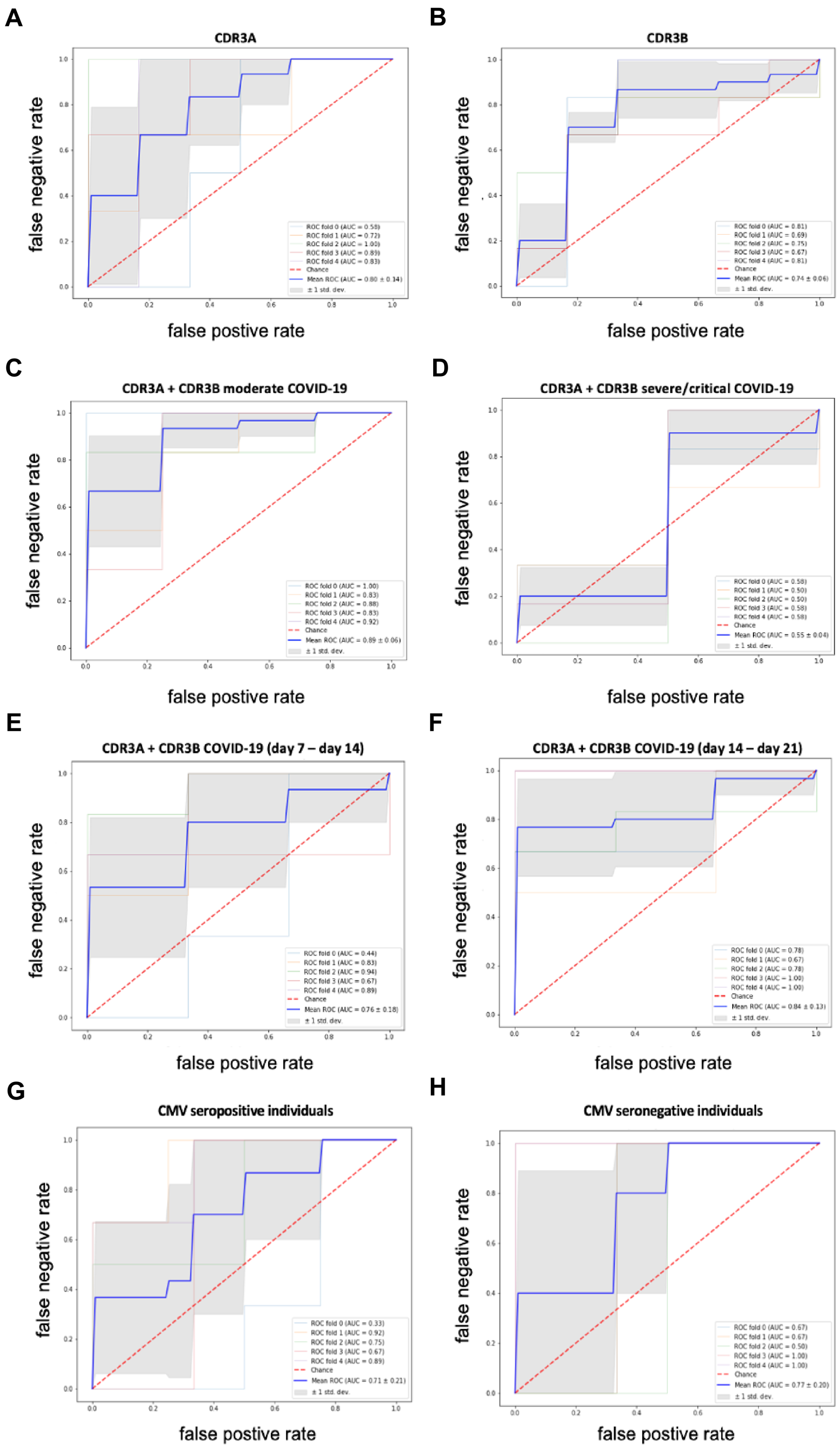

A
CDR3A
false negative rate
false postive rate
ROC fold 0 (AUC = 0.58)
ROC fold 1 (AUC = 0.72)
ROC fold 2 (AUC = 1.00)
ROC fold 3 (AUC = 0.89)
ROC fold 4 (AUC = 0.83)
Chance
Mean ROC (AUC = 0.80 ± 0.14)
± 1 std. dev.
B
CDR3B
false negative rate
false postive rate
ROC fold 0 (AUC = 0.81)
ROC fold 1 (AUC = 0.69)
ROC fold 2 (AUC = 0.75)
ROC fold 3 (AUC = 0.67)
ROC fold 4 (AUC = 0.81)
Chance
Mean ROC (AUC = 0.74 ± 0.06)
± 1 std. dev.
C
CDR3A + CDR3B moderate COVID-19
false negative rate
false postive rate
ROC fold 0 (AUC = 1.00)
ROC fold 1 (AUC = 0.83)
ROC fold 2 (AUC = 0.88)
ROC fold 3 (AUC = 0.83)
ROC fold 4 (AUC = 0.92)
Chance
Mean ROC (AUC = 0.89 ± 0.06)
± 1 std. dev.
D
CDR3A + CDR3B severe/critical COVID-19
false negative rate
false postive rate
ROC fold 0 (AUC = 0.58)
ROC fold 1 (AUC = 0.50)
ROC fold 2 (AUC = 0.50)
ROC fold 3 (AUC = 0.58)
ROC fold 4 (AUC = 0.58)
Chance
Mean ROC (AUC = 0.55 ± 0.04)
± 1 std. dev.
E
CDR3A + CDR3B COVID-19 (day 7 – day 14)
false negative rate
false postive rate
ROC fold 0 (AUC = 0.44)
ROC fold 1 (AUC = 0.83)
ROC fold 2 (AUC = 0.94)
ROC fold 3 (AUC = 0.67)
ROC fold 4 (AUC = 0.89)
Chance
Mean ROC (AUC = 0.76 ± 0.18)
± 1 std. dev.
F
CDR3A + CDR3B COVID-19 (day 14 – day 21)
false negative rate
false postive rate
ROC fold 0 (AUC = 0.78)
ROC fold 1 (AUC = 0.67)
ROC fold 2 (AUC = 0.78)
ROC fold 3 (AUC = 1.00)
ROC fold 4 (AUC = 1.00)
Chance
Mean ROC (AUC = 0.84 ± 0.13)
± 1 std. dev.
G
CMV seropositive individuals
false negative rate
false postive rate
ROC fold 0 (AUC = 0.33)
ROC fold 1 (AUC = 0.92)
ROC fold 2 (AUC = 0.75)
ROC fold 3 (AUC = 0.67)
ROC fold 4 (AUC = 0.89)
Chance
Mean ROC (AUC = 0.71 ± 0.21)
± 1 std. dev.
H
CMV seronegative individuals
false negative rate
false postive rate
ROC fold 0 (AUC = 0.67)
ROC fold 1 (AUC = 0.67)
ROC fold 2 (AUC = 0.50)
ROC fold 3 (AUC = 1.00)
ROC fold 4 (AUC = 1.00)
Chance
Mean ROC (AUC = 0.77 ± 0.20)
± 1 std. dev.

Supplementary figure 7: disentangling the contribution of common cold coronavirus cross-reactive TCR responses. The RefSeq genomes of the four common cold causing coronaviruses: HCoV-OC43, HCoV-HKU1, HCoV-229E and HCoV-NL63 were obtained from NCBI. NCBI BLAST was run on the 1205 SARS-CoV-2 epitopes from our TCR database against the four genomes. In total 52 epitopes were found to be shared by at least one of the common cold coronaviruses (allowing one mismatch between epitopes). **A.** Depth of SARS-CoV-2 associated TCRs when matched against the SARS-CoV-2 epitope-TCR database depleted for putative common cold (HCoV-OC43, HCoV-HKU1, HCoV-229E and HCoV-NL63) cross-reactive TCRs. **B.** Depth of putative common cold (HCoV-OC43, HCoV-HKU1, HCoV-229E and HCoV-NL63) cross-reactive TCRs. Of the thirty included patients, twenty had moderate, five severe, and five critical COVID-19. In the control group, ten individuals had past SARS-CoV-2 infection (exposed (exp)), ten had no evidence of a previous SARS-CoV-2 infection although being sampled during the pandemic (non-exposed-pandemic (non-exp-p)) and ten were non exposed controls sampled before the pandemic (non-exposed-pre-pandemic (non-exp-pp)). **C.** Common cold coronavirus depleted ROC by using SARS-CoV-2 associated TCR depth, after depletion of putative common cold coronavirus cross-reactive TCRs, in a logistic regression classifier. P-values denote student T-testing (non-significant (ns) $p > 0.05$, *** $p \leq 0.001$).

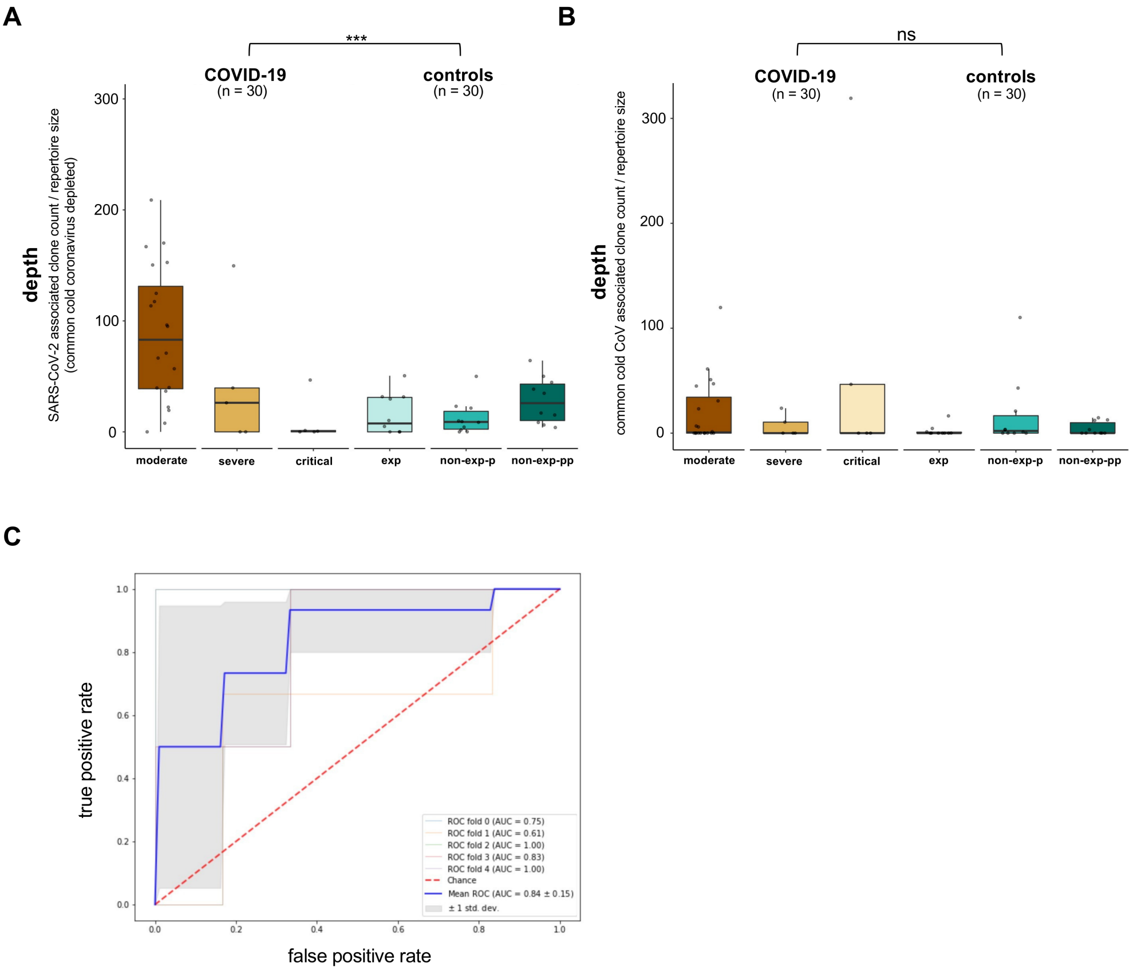

Supplementary figure 8: Comparison of whole blood lymphocyte and T cell counts between patients with moderate COVID-19 severity (n=20) and those with severe and critical COVID-19 combined (n=10). Four different T cell subsets, including overall lymphocyte count (lymf/µl) (panel A) CD3+ (panel B), CD4+ (panel C), CD8+ (panel D) were analyzed. P value denotes student T-testing (non-significant (ns) p > 0.05).

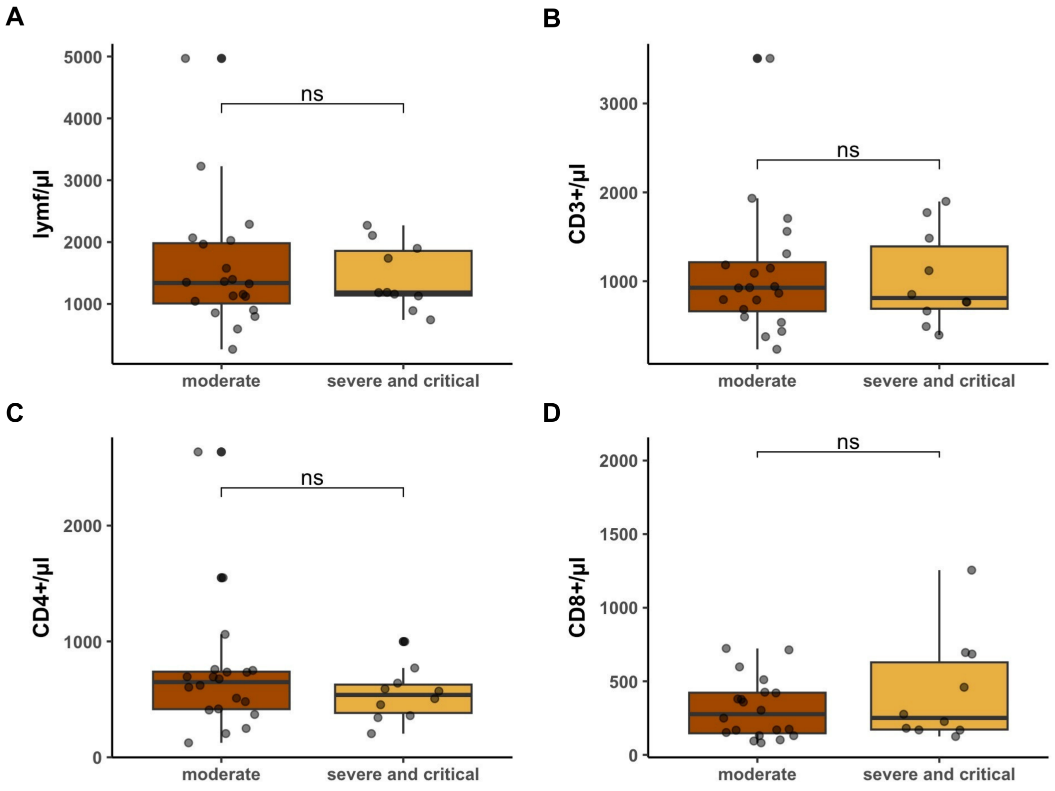

Supplementary table 1: Overview of sequenced samples.

								PBMC (flowcytometry)			whole blood counts			
disease severity	study_ID	age	sex	day from symptom onset	Wantai serology	activated subset sequenced	non-activated subset sequenced	% CD3+	% CD3+ CD8+	% CD3+/CD8+/ CD38+/HLADR+	total lymfocytes	CD3+ / µl	CD4+ / µl	CD8+ / µl
severe	IM002	66	M	10	positive	yes	yes	70,2	24,6	39,1	1898	1483	771	685
moderate	IM005	57	F	10	positive	yes	yes	70,4	18,6	1,43	1044	788	695	82
severe	IM008	81	M	18	positive	yes	yes	9,3	10,4	8,28	1183	770	571	169
critical	IM009	72	M	13	positive	yes	yes	10,3	12,4	12,1	892	394	204	168
moderate	IM012	36	F	9	positive	yes	yes	54,9	16,7	7,33	856	537	370	131
moderate	IM013	53	F	8	positive	yes	yes	81,4	10,8	8,48	1130	941	759	169
moderate	IM015	61	F	19	positive	yes	yes	73,5	32,7	19,4	2026	1182	750	380
moderate	IM020	56	M	19	positive	yes	yes	76,2	36,4	10,8	2068	1706	735	723
critical	IM021	60	F	7	negative	yes	yes	67,4	15,4	6,67	1187	852	640	181
moderate	IM022	65	M	16	positive	yes	yes	54,7	25,6	1,51	1154	683	408	250
critical	IM024	50	M	20	positive	yes	yes	69,6	22	6,55	1129	766	454	226
critical	IM025	43	M	12	positive	yes	yes	20,4	21,3	12,6	1159	666	359	276
moderate	IM027	55	F	7	positive	yes	yes	75,3	13,7	1,79	1122	864	694	152
severe	IM028	35	M	12	positive	yes	yes	78	35,8	16,8	2108	1771	999	695
moderate	IM029	66	F	17	positive	yes	yes	59	16,7	15,3	2288	1562	1061	426
moderate	IM031	50	M	14	positive	yes	yes	77,8	29,7	7,27	1577	1309	734	511
moderate	IM032	54	M	19	positive	yes	yes	74,6	47,6	6,21	1397	1149	510	598
severe	IM033	61	F	15	positive	yes	yes	43,8	38,3	8,69	1738	1118	590	459
moderate	IM035	58	M	13	positive	yes	yes	59,9	34,2	12,5	1362	929	480	421
moderate	IM036	50	F	15	positive	yes	yes	60,1	17,8	14,3	4969	3507	2636	713
critical	IM038	75	M	18	positive	yes	yes	60	15,6	15,4	743	491	342	125
moderate	IM039	41	M	12	positive	yes	yes	25,7	16,9	8,51	901	599	418	168
moderate	IM045	57	M	11	positive	yes	no	41,7	28,7	4,77	595	376	249	102
moderate	IM047	63	M	18	positive	yes	yes	56,2	15,5	11,5	798	434	205	174
moderate	IM049	30	F	15	positive	yes	yes	74,8	32,5	15,2	1351	1092	677	377
moderate	IM050	54	F	18	positive	yes	no	45,4	27,5	5,06	1967	925	605	303
severe	IM053	46	M	8	negative	yes	yes	76,6	56,1	7,66	2271	1897	506	1255
moderate	IM055	56	F	14	positive	yes	no	55,1	21	9,15	3226	1933	1550	359
moderate	IM059	61	M	8	negative	yes	no	64	19,1	6,08	1326	792	621	130

moderate	IM060	71	M	9	negative	yes	yes	52,7	18,5	20,4	267	234	125	94
exposed controls	IM015	61	F	336	positive	yes	yes	76,7	35	4,89	NaN	NaN	NaN	NaN
exposed controls	IM029	66	F	164	positive	yes	yes	70,9	23,7	1,79	NaN	NaN	NaN	NaN
exposed controls	IM053	46	M	80	positive	yes	yes	78,7	61,2	5,81	NaN	NaN	NaN	NaN
exposed controls	IM002	66	M	332	positive	yes	yes	52,8	38,2	8,75	NaN	NaN	NaN	NaN
exposed controls	IM021	60	F	308	positive	yes	yes	67,8	16,6	0,96	NaN	NaN	NaN	NaN
exposed controls	IMC04	41	F	136	positive	yes	yes	71	21	0,98	NaN	NaN	NaN	NaN
exposed controls	IMC07	61	F	98	positive	yes	yes	69,8	26,1	2,29	NaN	NaN	NaN	NaN
exposed controls	IMC14	62	M	297	positive	yes	yes	70,3	18,5	7,3	NaN	NaN	NaN	NaN
exposed controls	IMC33	58	M	85	positive	yes	yes	66,4	20	2,02	NaN	NaN	NaN	NaN
exposed controls	IMC34	39	M	344	negative	yes	yes	76,4	48,7	6,5	NaN	NaN	NaN	NaN
non exposed pandemic	IMC01	52	F	NaN	negative	yes	no	70,8	28,9	0,66	NaN	NaN	NaN	NaN
non exposed pandemic	IMC03	57	F	NaN	negative	yes	no	78,3	28,2	1,42	NaN	NaN	NaN	NaN
non exposed pandemic	IMC05	41	F	NaN	negative	yes	no	80,5	12,9	1,52	NaN	NaN	NaN	NaN
non exposed pandemic	IMC08	43	F	NaN	negative	yes	no	78,5	30,8	1,29	NaN	NaN	NaN	NaN
non exposed pandemic	IMC10	49	M	NaN	negative	yes	no	63,4	37,9	2,06	NaN	NaN	NaN	NaN
non exposed pandemic	IMC15	42	M	NaN	negative	yes	no	74	32,1	5	NaN	NaN	NaN	NaN
non exposed pandemic	IMC21	47	M	NaN	negative	yes	no	68,2	33,1	3,05	NaN	NaN	NaN	NaN

non exposed pandemic	IMC22	28	F	NaN	negative	yes	no	61,6	25,6	5,26	NaN	NaN	NaN	NaN
non exposed pandemic	IMC23	58	M	NaN	negative	yes	no	74	20,1	2,88	NaN	NaN	NaN	NaN
non exposed pandemic	IMC28	26	M	NaN	negative	yes	no	63,7	23,4	1,66	NaN	NaN	NaN	NaN
non exposed pre pandemic	MMR21	28	F	NaN	NaN	yes	no	62,7	26,3	2,03	NaN	NaN	NaN	NaN
non exposed pre pandemic	MMR25	31	M	NaN	NaN	yes	no	64,9	34,2	3,33	NaN	NaN	NaN	NaN
non exposed pre pandemic	MMR27	32	M	NaN	NaN	yes	no	70,3	26,8	3,93	NaN	NaN	NaN	NaN
non exposed pre pandemic	MMR28	26	F	NaN	NaN	yes	no	68,1	36,4	3,02	NaN	NaN	NaN	NaN
non exposed pre pandemic	MMR37	29	F	NaN	NaN	yes	no	81,5	21,6	2,09	NaN	NaN	NaN	NaN
non exposed pre pandemic	MMR38	27	M	NaN	NaN	yes	no	67,1	37,3	0,94	NaN	NaN	NaN	NaN
non exposed pre pandemic	MMR5	28	F	NaN	NaN	yes	no	63,4	20,6	1,7	NaN	NaN	NaN	NaN
non exposed pre pandemic	MMR7	34	F	NaN	NaN	yes	no	54,2	39,5	2,12	NaN	NaN	NaN	NaN
non exposed pre pandemic	MMR8	28	F	NaN	NaN	yes	no	62,9	26,7	2,07	NaN	NaN	NaN	NaN
non exposed pre pandemic	MMR9	26	M	NaN	NaN	yes	no	56,1	39,2	1,03	NaN	NaN	NaN	NaN

Supplementary table 2: Summary of WHO criteria: COVID-19 disease severity in adults.

**Mild**	**Symptomatic COVID-19 with:**	**No clinical evidence of viral pneumonia* or hypoxia.**
**Moderate**	Clinical evidence of pneumonia* with:	RR ≤ 30 breaths/minute **AND** SpO_2 ≥ 90% on room air
**Severe**		RR > 30 breaths/minute **OR** SpO_2 < 90% on room air **OR** clinical signs of severe respiratory distress
**Critical**	ARDS with:	PaO_2/FiO_2 ≤ 300 mmHg with PEEP or CPAP ≥ 5 cmH_2O **OR** need for invasive ventilation

*Clinical evidence of pneumonia: fever, cough, dyspnea, fast breathing
